# Enhancing the oxidation of polystyrene through a homogeneous liquid degradation system for effective microbial degradation

**DOI:** 10.3389/fmicb.2024.1509603

**Published:** 2024-11-28

**Authors:** Hong Rae Kim, Hye Yeon Koh, Hyeyoung Shin, Dong-Eun Suh, Sukkyoo Lee, Donggeon Choi

**Affiliations:** ^1^Department of Research and Development, Repla Inc., Suwon, Republic of Korea; ^2^Department of Brain Sciences, Daegu Gyeonbuk Institute of Science and Technology, Daegu, Republic of Korea

**Keywords:** homogeneous reaction, *Pseudomonas aeruginosa*, plastic, microbial biodegradation, liquid-state degradation, microplastics

## Abstract

Plastics play a crucial role in modern industries; however, their resistance to natural degradation contributes to environmental pollution, and microplastics pose a health threat. The hydrophobic nature of microplastics poses a considerable challenge, rendering them resistant to dissolving in water. In this study, we conducted a comparative analysis of the microbial biodegradation capabilities of polystyrene in solid and liquid states. Polystyrene in its solid foam form, along with polystyrene converted into a liquid state using ethyl-ester oil, was biodegraded by microorganisms. Subsequently, the liquid plastic was re-extracted into its solid form, and the degree of degradation was assessed using weight loss measurement, XPS, FT-IR, GPC, and TGA. Liquid-state polystyrene exhibited a higher degradation rate than that reported previously. Furthermore, liquid polystyrene undergoes more pronounced oxidation than its solid counterpart, leading to an increased oxygen atom ratio. Chemical structure analysis highlighted the distinct formation of –OH and C=O functional groups in the liquid state compared to those in the solid state. Additionally, notable changes in the molecular weight and thermal stability of polystyrene were observed during biodegradation in the liquid state. This study suggests that a heterogeneous reaction (solid plastic-liquid medium) might impede plastic biodegradation, while indicating the potential to enhance the degradation efficiency through a homogeneous reaction (liquid plastic-liquid medium). The follow-up study identifies appropriate solvents and optimizes cultivation conditions, offering potential to enhance the efficiency of biological plastic degradation.

## 1 Introduction

Plastics are widely used in modern industries owing to their affordability, light weight, ease of processing, and durability. Despite these advantages, the natural degradation rate of plastics is exceedingly slow and it is estimated to take approximately 500 years for complete degradation to occur. When exposed to the environment, plastic wastes become microplastics, which contaminate soil and water bodies, and have been implicated in endocrine system disorders and various diseases (MacLeod et al., 2021). Two-thirds of all plastic has a short life cycle of less than 1 month, and after use, it is primarily disposed of through landfilling or incineration, contributing significantly to environmental pollution. In response, the United Nations Environment Programme (UNEP) is working to address the issue of microplastics in oceans and soils by reducing plastic waste usage and improving waste management methods. The European Union (EU) has also implemented legislation to reduce plastic pollution, while individual countries are encouraging investment in recycling initiatives and the development of waste management technologies to address the pollution crisis (Johansen et al., 2022; Pilapitiya and Ratnayake, 2024).

Plastics are diverse and can be classified as polyesters, polyamides, or polyolefins based on their characteristics. Polyesters, with monomers connected by ester bonds, are easier to depolymerize, whereas polyolefins, composed of carbon–carbon bonds, pose challenges in degradation (Ali et al., 2021). The most widely used plastics, including polyethylene (PE), polypropylene (PP), polystyrene (PS), polyvinyl chloride (PVC), polyethylene terephthalate (PET), and polyurethane (PUR), collectively account for 91% of the total plastic usage. Excluding PET (7%) and PUR (6%), polyolefin-based plastics—PE (32%), PP (23%), PS (7%), and PVC (16%)—all featuring a C-C backbone, present a substantial challenge in addressing plastic waste disposal issues. The development of effective degradation methods for polyolefin plastics is therefore crucial (Ali et al., 2021; Zhang et al., 2022). Microbial degradation has recently emerged as a promising approach for plastic waste management. Microorganisms provide an environmental friendly solution for degrading plastics at room temperature and atmospheric pressure (Tournier et al., 2023). Ongoing research is focused on identifying microorganisms capable of degrading plastics in various environments, including the digestive systems of insects, marine ecosystems, and landfills. For instance, *Gordonia* sp. achieved 7.73% polystyrene degradation over 30 days and *Exiguobacterium* sp. achieved 7.4% polystyrene degradation over 28 days (Yang et al., 2015; Kim et al., 2021; Liu et al., 2023). These degradation rates are insufficient for industrial applications, highlighting the need to improve the efficiency. Previous studies have focused on identifying efficient microorganisms or microbial communities capable of degrading plastics and elucidating microbial metabolic pathways to improve the degradation rate (Zadjelovic et al., 2022). However, the challenge of plastic degradation also stems from the inherent material properties of plastics (Thew et al., 2023). Therefore, in this study, the efficiency of microbial degradation of PS was explored with a focus on the plastic’s characteristics.

Two primary challenges impede the effective degradation of plastics using microorganisms. First, the substantial molecular weight of plastics makes it difficult for microorganisms to transport them into their cells. Second, the hydrophobic nature of plastic molecules hinders biofilm formation and enzyme reactions (Tokiwa et al., 2009; Yasin et al., 2022). Taking a closer look at the second challenge, as plastics do not dissolve in water, microbial and enzymatic reactions are confined to the surface of solid-state plastics. This limitation plays a role in slowing the degradation process. Typical chemical reactions proceed swiftly in a homogeneous system in which the substrate and solvent share the same state. However, in heterogeneous systems, such as when a solid substrate reacts with a liquid solvent, as observed for plastics, the reaction rate is significantly diminished. From this perspective, previous studies have explored the increase in biodegradation efficiency when plastics in the solid state are heated above the glass transition temperature, resulting in the weakening of intermolecular bonds (Tokiwa et al., 2009; Maurya et al., 2020). However, the effect of transforming plastics into a liquid state on their biodegradation has not yet been analyzed. Beyond loosening the intermolecular interactions of plastics in their solid state, it is necessary to analyze the impact of separating them into individual molecules and liquefying them on biological degradation. This approach will enable a more detailed understanding of how the material properties of plastics influence the degradation process.

In our study, we aimed to verify the hypothesis that the resistance of plastics to dissolving in water, which forces them to react in a solid state, acts as one of the rate-limiting factors for their degradation. To investigate this, we transformed PS, a polyolefin-based plastic, into a liquid state and cultivated microorganisms in a homogeneous system to assess improvements in degradation capabilities. Additionally, we analyzed the proteins whose expression levels were elevated in response to liquid PS, identifying potential enzymes involved in plastic degradation. This approach enabled the identification of how the material properties of plastics affect the rate of biological degradation and provided insights into potential strategies for enhancing degradation efficiency.

## 2 Materials and methods

### 2.1 Biodegradation of PS in solid and liquid phases

*Pseudomonas aeruginosa*, known for its ability to degrade PS, was employed to degrade PS in both the solid and liquid phases (Kim et al., 2020). The strain used in this study was derived from a species isolated from the gut of superworms that had ingested polystyrene, as identified in a previous study conducted by our research team (Lee et al., 2020). The liquid carbon-free basal medium (LCFBM) was composed of 0.7 g KH_2_PO_4_, 0.7 g K_2_HPO_4_, 0.7 g MgSO_4_·7H_2_O, 1 g NH_4_NO_3_, 0.005 g NaCl, and 0.002 g FeSO_4_·7H_2_O per 1 L. Additionally, 1 mL of a 100-fold trace mineral stock solution (0.3 g H_3_BO_3_, 0.2 g CoCl_2_·6H_2_O, 0.1 g ZnSO_4_·7H_2_O, 0.03 g MnCl_2_·4H_2_O, 0.03 NaMoO_4_·2H_2_O, 0.02 g NiCl_2_·6H_2_O, and 0.01 g CuSO_4_·5H_2_O per 1 L) was added to 1 L of LCFBM (Yang et al., 2015; Kim et al., 2020). Each solution was prepared separately and then mixed in the correct proportions before cultivation. Contamination was prevented by autoclaving the solutions individually. *P. aeruginosa* was precultured in Luria Bertani (LB) medium, followed by centrifugation (13,000 rpm, 5 min) and resuspension of the pellet in LCFBM. This process was repeated three times to remove all the nutritional components. The culture was then inoculated into 25 mL of LCFBM at a 1/1000 dilution based on the optical density. PS foam (Mw ~245,000 Da) was cut into 2 × 2 cm pieces, and 0.05 g of the plastic was used in the experiment. For the liquid plastic cultivation system, 0.05 g of PS foam was dissolved in 1 mL ethyl ester oil (a mixture of eicosapentaenoic and docosahexaenoic acids at a ratio of 10:7) (SOLGAR, Leonia, NJ, United States). The resulting solution was mixed with 25 mL of LCFBM and cultured at 28°C with aeration (130 rpm) for 7 days. Each sample was prepared in triplicate for each experimental group, and statistical significance was assessed using Student’s *t*-test. The significance level for the analysis was set at a *p*-value of less than 0.05.

### 2.2 PS recovery and degradation rate measurement

The degradation of PS was determined by measuring its weight reduction. Following cultivation, a 30-min settling period was introduced to facilitate the separation of the oil and LCFBM medium layers. The medium was then retrieved using a 50 mL syringe. For solid-state plastics, the material was air-dried at room temperature (20°C) for 24 h, and its weight was subsequently measured. PS dissolved in oil was recovered by precipitation with a mixture of methanol following the method described in a previous study (García-Barrera et al., 2022). The precipitate was washed with isopropanol to remove the residual oil. The recovered PS in solid form was air-dried at room temperature for 24 h, and its weight was measured.

### 2.3 Characterization of degraded PS

The chemical characteristics of the recovered PS were analyzed under various conditions, including solid-state controls, samples cultured with *P. aeruginosa* in the solid state, liquid-state controls, and samples cultured with *P. aeruginosa* in the liquid state (Wu and Criddle, 2021). The solid-state control involved incubating solid polystyrene in a medium without microorganisms under identical conditions. The liquid-state control involved incubating the liquefied polystyrene in a medium without microorganisms under the same conditions. X-ray photoelectron spectroscopy (XPS) (K-Alpha, Thermo Fisher Scientific, Waltham, MA, United States) was conducted to analyze the elemental composition and binding energy on the surface of the polystyrene. The samples were affixed with carbon tape and analyzed within an energy range of 276–300 eV for the C1s scan and 0–1,350 eV for the survey scan (Kim et al., 2020). To identify changes in the chemical structure of PS, attenuated total reflectance-Fourier transform infrared spectroscopy (ATR-FTIR) spectrometer (iS50, Thermo Fisher Scientific, Waltham, MA, United States) was employed to observe alterations in the functional groups. ATR-FTIR analysis used single-bounce attenuated total reflection spectroscopy with wavenumbers ranging from 400 cm^−1^ to 4,400 cm^−1^ (Yang et al., 2015).

Molecular weight analysis of PS was conducted through gel permeation chromatography (GPC). PS samples, dissolved in tetrahydrofuran (THF) at a concentration of 3 mg/mL, were filtered through a 0.45 μm polytetrafluoroethylene (PTFE) filter. The solution (10 μL) was measured at a flow rate of 0.35 mL/min and at a temperature of 40°C using GPC (Tosoh EcoSEC HLC-8420 GPC, Tosoh Bioscience, Tokyo, Japan). Thermogravimetric analysis (TGA) was performed to assess the thermal stability of PS. Approximately 100 mg of the sample was heated from room temperature to 600°C at a rate of 20°C /min. The analysis was conducted under nitrogen at a flow rate of 100 mL/min using a thermogravimetric analyzer (TGA Q5000 IR, TA Instruments, New Castle, DE, United States) (Nyamjav et al., 2023).

### 2.4 Protein expression analysis of *Pseudomonas aeruginosa* in the presence of PS

The culture broth was centrifuged to separate the cell pellet and supernatant. The supernatant, containing the soluble protein fraction was collected after centrifugation at 13,000 rpm for 30 min at 4°C to remove cell debris containing insoluble proteins. The cell debris was disrupted by ultrasonication on ice during 5 cycles of 10 s pulses with 30 s intervals and 30% amplitude using an ultrasonic cell disruptor (Q500 Sonicator, Qsonica Llc, Newtown, CT, United States), centrifuged at 13,000 rpm for 5 min at 4°C and separated into the pellet and cytoplasm. Proteins were extracted from each fraction, and their concentrations were determined using the Bradford method (Sigma-Aldrich, St. Louis, MO, United States) with bovine serum albumin as a standard (Thermo Fisher Scientific, Waltham, MA, United States) (Kruger, 2009). The proteins were then boiled with a protein-loading dye. One-dimensional sodium dodecyl sulfate-polyacrylamide gel electrophoresis (SDS-PAGE) was performed using 4–20% gradient acrylamide gels. Protein bands were visualized using Coomassie Blue R-250 staining (Bio-Rad Laboratories, Hercules, CA, United States) (Mazur et al., 2023). *P. aeruginosa* cultured in ethyl ester oil with no PS dissolved, was used as a control. Bands exhibiting differences were subjected to protein profiling using LC–MS protein sequencing (Supplementary Text S1).

## 3 Result and discussion

### 3.1 Improving degradation rates through PS liquefaction

**Figure 1:**
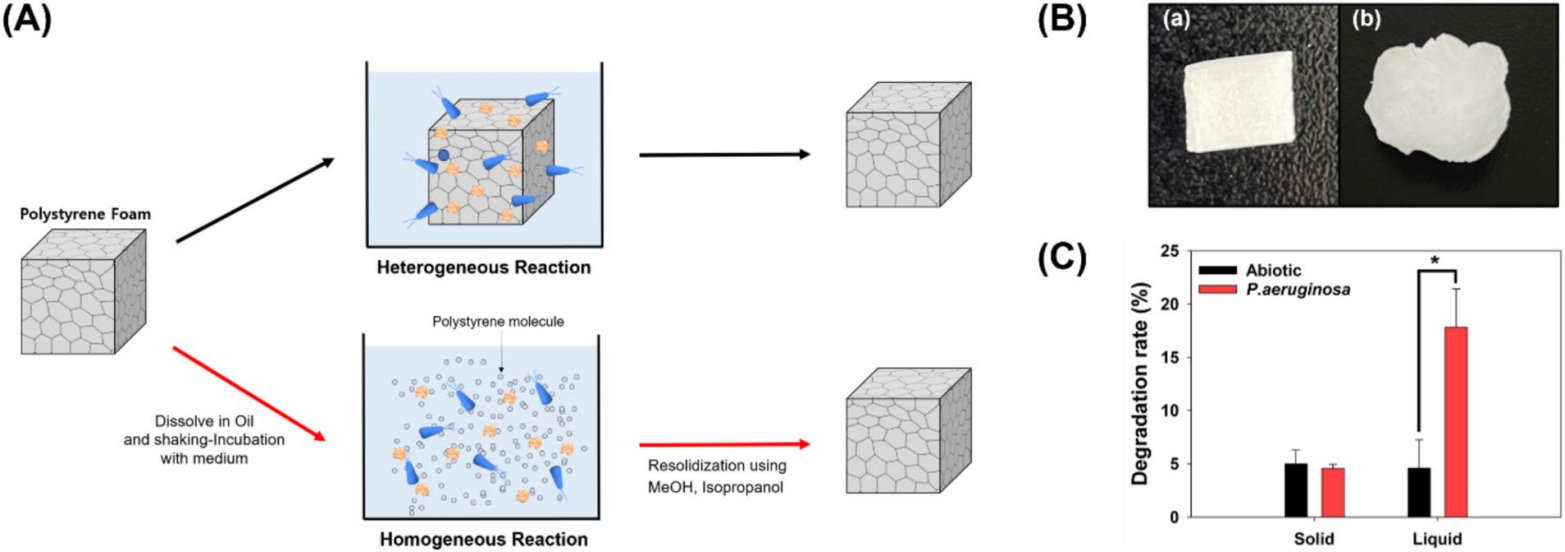
Heterogeneous and homogeneous biodegradation of polystyrene. **(A)** Overview of the experimental process: Polystyrene foam is dissolved in oil, transitioned into a liquid state, and subjected to microbial reactions. **(B)** Types of polystyrene used in the experiment. (a) Polystyrene reacted with microorganisms in the solid state. (b) Polystyrene reacted with microorganisms in the liquid state and subsequently solidified. **(C)** Degradation rates of polystyrene treated in solid and liquid states.

In this study, solid-state plastics were transformed into the liquid state and reacted with *P. aeruginosa* to facilitate the biodegradation of PS (Kim et al., 2020). Solid-state plastics only undergo enzymatic and microbial reactions on their surfaces. Conversely, in the liquid state, each PS molecule interacts with enzymes and microorganisms, potentially leading to an accelerated degradation rate (Figure 1). To validate this hypothesis, PS was dissolved in ethyl ester oil, exposed to microorganisms for 7 days, and subsequently recovered to analyze its degradation rate and characteristics of the degraded PS (Figures 1B,C).

The degradation rates of solid and liquid PS were measured, and no observable weight loss occurred in the solid-state PS compared to that in the control group (Figure 1C). Degradation of solid-state plastics occurs only on the surface, resulting in a very slow rate of weight loss. In fact, when *P. aeruginosa* was provided with solid-state PS foam as a carbon source and cultured, it successfully proliferated using PS (Supplementary Table S1). However, the degradation rate was not significant enough to be confirmed using weight loss measurements. In contrast, in cultivations with the liquid state polystyrene, PS exhibited a weight loss of 13.24% after 7 days. The plastic weight loss by microorganisms was calculated to be 13.24%, based on the difference between the experimental group (17.83%) and the control group (4.59%). The degradation rate observed in this study surpassed that reported in the literature. For example, *Gordonia* sp. achieved a degradation rate of 7.73% over 30 days for foam-type solid polystyrene, and *Exiguobacterium* sp. achieved 7.40% over 28 days for film-type solid polystyrene (Yang et al., 2015; Liu et al., 2023).

In previous studies, a liquid carbon-free basal medium (LCFBM) was used for microbial cultivation. Research involving *Exiguobacterium* sp. utilized approximately 0.05 g of foam-type polystyrene, while studies with *Gordonia* sp. used film-type polystyrene. In this study, LCFBM was also employed, and 0.05 g of foam-type polystyrene was used, making the experimental materials nearly identical. While previous studies assessed the plastic degradation ability of novel microorganisms by measuring the weight loss of solid-state polystyrene, this study evaluated degradation after converting polystyrene into a liquid form.

The process by which microorganisms degrade plastics typically occurs in three stages. The first stage, biodeterioration, involves microorganisms transforming the chemical groups on the plastic’s surface. To increase the hydrophilicity of the plastic, oxidative enzymes introduce oxygen atoms into the material. In the second stage, biofragmentation, the oxidized plastic undergoes a reduction in molecular weight, and microbial enzymes break it down into oligomers. Finally, in the third stage, these products are mineralized through the microorganisms’ internal metabolic pathways (Ali et al., 2021; Zhang et al., 2022). Therefore, the efficiency of microbial enzymes and their interactions with plastic are critical for effective degradation. When degrading solid plastic, enzymatic reactions are limited to the surface molecules of the material. However, when the plastic is liquefied, individual molecules that were previously within the plastic matrix can also react simultaneously. This increased availability of molecules for enzymatic action suggests that liquefying polystyrene can enhance degradation efficiency. To accurately evaluate the reaction efficiency between plastic molecules and enzymes, we analyzed the incorporation of oxygen atoms and the formation of chemical groups such as C-O, C=O, and –OH in the first stage of microbial plastic degradation (Kim et al., 2023). Additionally, we examined the changes in the molecular weight of the plastic during the second stage of degradation.

### 3.2 Characterization of biodegraded PS

**Figure 2:**
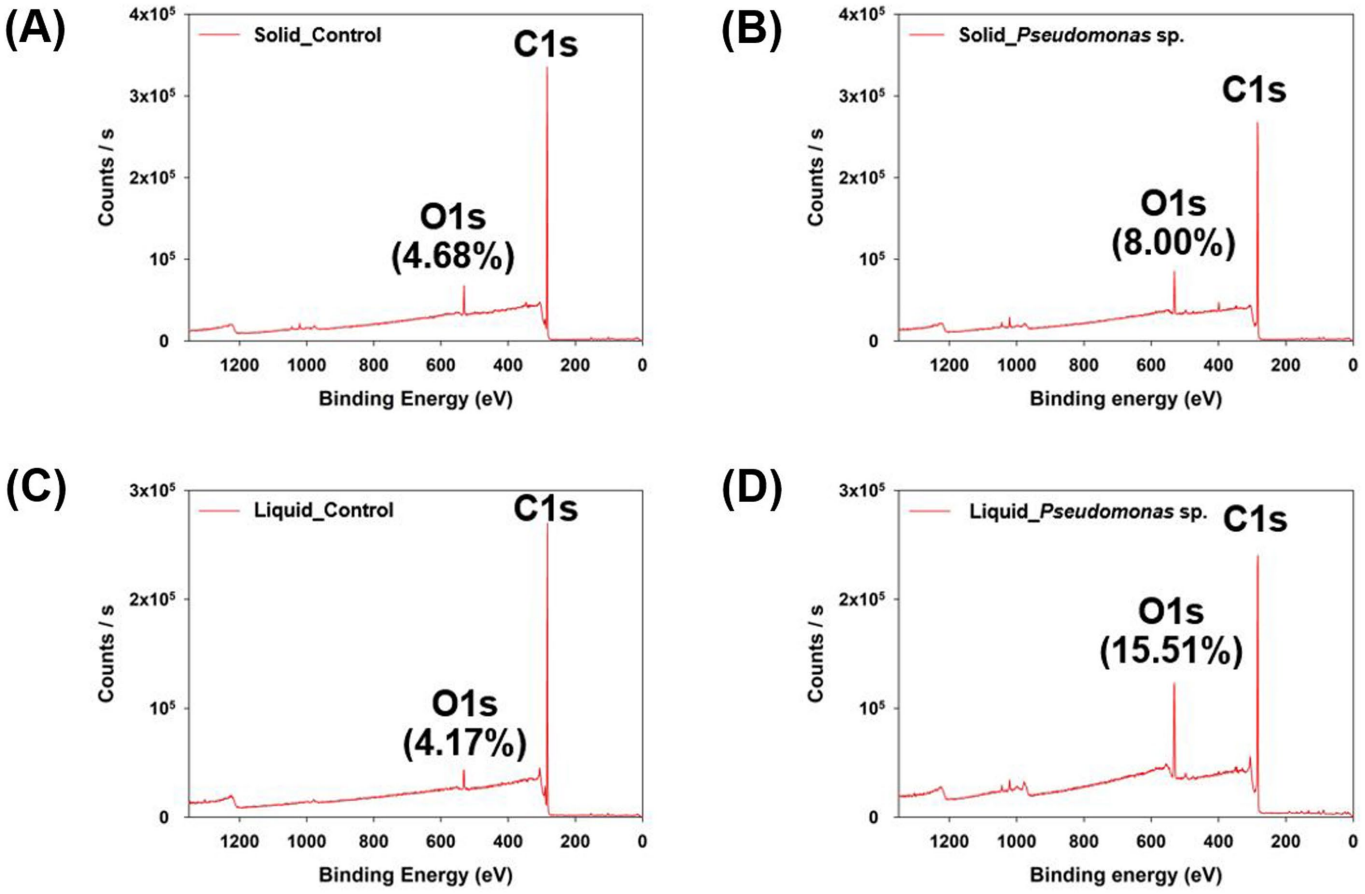
Elemental analysis of polystyrene reacted in solid and liquid phases using XPS. **(A)** Control group cultured with *P. aeruginosa* in the solid state. **(B)** Polystyrene cultured with *P. aeruginosa* in the solid state. **(C)** Control group cultured with *P. aeruginosa* in the liquid state. **(D)** Polystyrene cultured with *P. aeruginosa* in the liquid state.

Subsequently, the characteristics of biodegraded PS were analyzed. First, the surface elements were analyzed using XPS (Supplementary Table S2). After the experiment in the solid and liquid states, the PS control samples exhibited oxygen element ratios of 4.68 and 4.17%, respectively (Figures 2A,C). The PS control sample refers to the group that was incubated in the medium without microorganisms. However, when reacted with *P. aeruginosa*, the samples exhibited an oxygen element ratio of 8.00 and 15.51% in the solid state and liquid state reactions, respectively (Figures 2B,D). This indicates that oxidation reactions occurred on the PS surface owing to the interaction with *P. aeruginosa*, with more pronounced oxidation observed when reacted in the liquid phase. The first stage of plastic degradation by microorganisms involves surface oxidation and hydrophilization facilitated by oxidative enzymes. Plastics are highly hydrophobic, which makes them poorly soluble in water and less reactive with enzymes. As a result, oxidative enzymes introduce oxygen atoms in the form of hydroxyl groups (–OH), and functional groups such as C-O or C=O are formed through the action of alcohol dehydrogenase and aldehyde dehydrogenase (Hou and Majumder, 2021; Kim et al., 2023). The increase in oxygen atom ratio observed in the surface elemental analysis of the plastic indicates that oxidative enzymes produced by microorganisms reacted with the plastic. This suggests that the reaction was more efficient in the liquid state.

**Figure 3:**
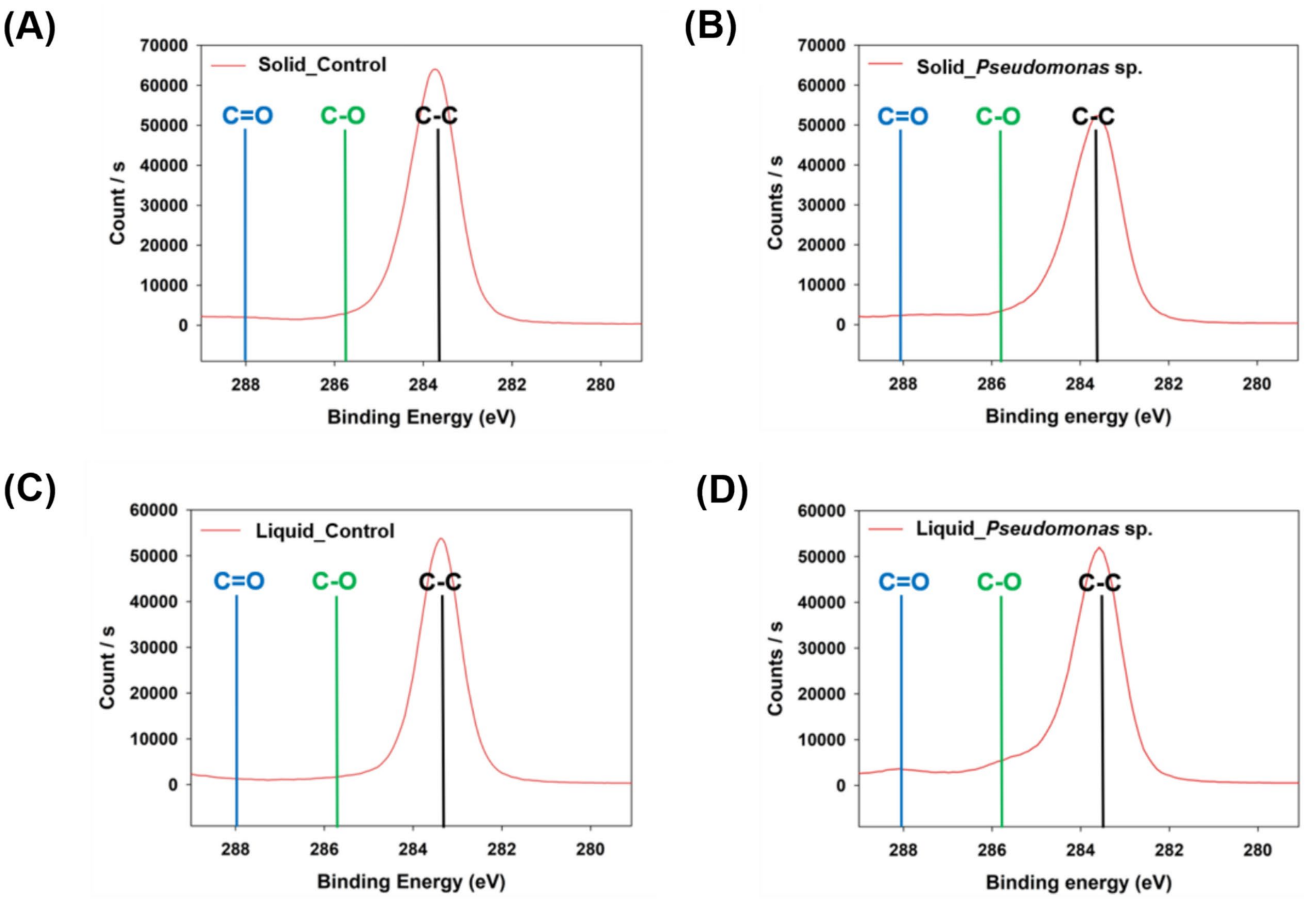
Analysis of carbon binding energy in polystyrene using XPS. **(A)** Control group cultured with *P. aeruginosa* in the solid state. **(B)** Polystyrene cultured with *P. aeruginosa* in the solid state. **(C)** Control group cultured with *P. aeruginosa* in the liquid state. **(D)** Polystyrene cultured with *P. aeruginosa* in the liquid state.

**Figure 4:**
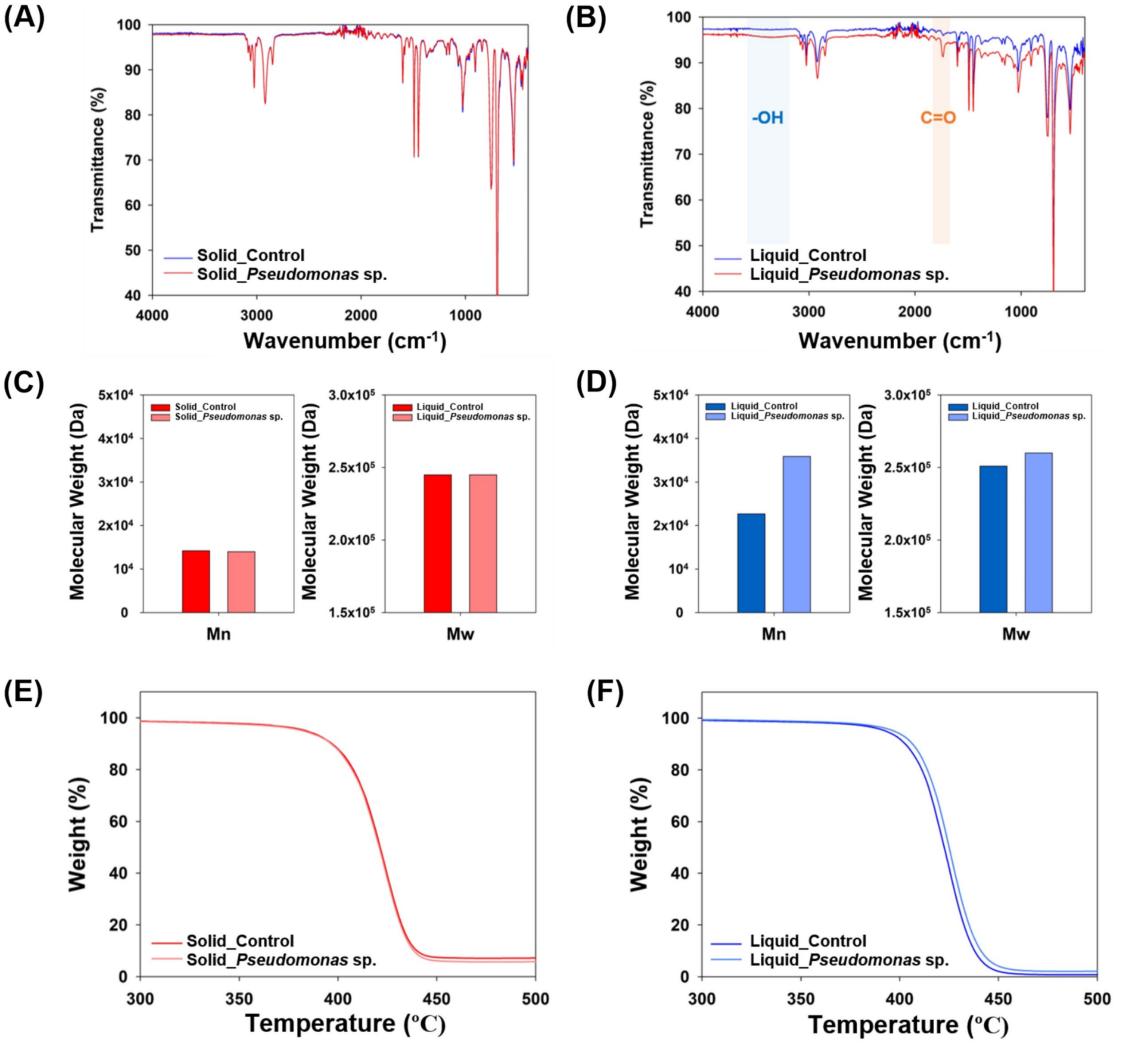
Characterization of degraded polystyrene. **(A)** FT-IR measurement results for polystyrene experimented in its solid state. **(B)** FT-IR measurement results for polystyrene experimented in its liquid state. **(C)** Molecular weight of polystyrene experimented in its solid state. **(D)** Molecular weight of polystyrene experimented in its liquid state. **(E)** TGA results for polystyrene experimented in its solid state. **(F)** TGA results for polystyrene experimented in its liquid state.

Additionally, the analysis of the carbon binding energy indicated that no C-O or C=O bonds were detected in the control group (Figures 3A,C and Supplementary Figures S1A,C). However, when *P. aeruginosa* was reacted in the solid state, a weak C=O bond was observed (Figure 3B and Supplementary Figure S1B). In the liquid-state reaction, both C-O and C=O bonds were distinctly observed (Figure 3D and Supplementary Figure S1D), and the formation of C-O and C=O bonds is considered as the evidence for PS oxidation, which represents the first step in the biodegradation process (Yang et al., 2015; Kim et al., 2020). To further validate these findings, FT-IR analysis was conducted. No significant differences were observed in the peaks when the reaction occurred in the solid-state between experimental and control samples (Figure 4A). In the cultivation experiment with *P. aeruginosa*, an increase in oxygen was observed in XPS analysis. However, functional groups were not detected in the FTIR analysis. This discrepancy may be attributed to the higher precision of XPS results compared to that of FT-IR measurements. In contrast, when the reaction occurred in the liquid state, distinct peaks corresponding to hydroxyl groups (–OH) and carbonyl groups (C=O) on PS induced by *P. aeruginosa* were observed (Figure 4B). The presence of a –OH peak at approximately 3,300 cm^−1^ and a C=O peak at approximately 1,700 cm^−1^ provides indicative evidence of PS oxidation (Yang et al., 2014; Kim et al., 2020). This suggests that microbial-induced oxidation of PS is more effective in the liquid state.

Analysis of the molecular weight of degraded PS using GPC revealed no significant difference when solid-state PS was incubated with *P. aeruginosa* compared to that of the control group (Figure 4C). In contrast, when cultured in the liquid state, both the number average molecular weight (M_n_) and weight average molecular weight (M_w_) increased by 58.2 and 3.6%, respectively, in samples cultured with *P. aeruginosa* (Figure 4D). The increase in the molecular weight of plastics following microbial cultivation indicates more efficient degradation of low-molecular-weight plastics (Kawai et al., 2004; Peng et al., 2020; Yang et al., 2021). When the depolymerization of plastics occurs, both the number-average molecular weight (M_n_) and weight-average molecular weight (M_w_) typically decrease. However, if microorganisms rapidly degrade low molecular weight compounds, an overall increase in molecular weight may occur. The larger fluctuation in M_n_, which is more influenced by low molecular weight substances, compared to M_w_, which is more affected by high molecular weight substances, supports this finding. Therefore, it appears that the *P. aeruginosa* strain is capable of degrading low molecular weight polystyrene (PS), but its ability is limited at higher molecular weights.

Additionally, TGA was conducted using the degraded PS. In the solid state, no significant difference was observed between the control group and the cultured group with *P. aeruginosa* (Figure 4E). However, under liquid state experimental conditions, *P. aeruginosa* group showed improved thermal stability from 430°C to 432°C (Figure 4F). An increase in the molecular weight of polymers due to microbial reactions can enhance thermal stability (Calahorra et al., 1989). Since thermal stability is closely linked to the polymer’s molecular weight, the increase in molecular weight observed in the GPC results was further confirmed by the TGA data (Kokta et al., 1973). These changes in the characteristics of the plastics induced by microorganisms were more pronounced when the plastics were in their liquid state.

### 3.3 Changes in microbial protein expression induced by PS

Microbial protein expression patterns induced by liquid-state PS were analyzed. Compared to *P. aeruginosa* cultured with ethyl ester oil, *P. aeruginosa* proliferated with PS dissolved in ethyl ester oil, revealing a new protein band (Supplementary Figure S2). In the supernatant, a new band was observed at approximately 30 kDa, whereas in the cytoplasm, a distinct band was observed at 60 kDa. To elucidate the function of this protein, protein sequencing and profiling were performed. The specific enzymes involved in polystyrene degradation by microorganisms have not been thoroughly studied. Therefore, the overexpressed enzymes in this study could serve as potential candidates for involvement in plastic degradation.

In the new band observed in the cytoplasm, four major proteins were identified (Table 1 and Supplementary Table S3). Catalase functions as an enzyme that decomposes hydrogen peroxide into water and oxygen, playing a role in degrading reactive oxygen species within the cell (Cabiscol et al., 2000). The plastic degradation process involves oxidation reactions with the participation of reactive oxygen species (Zadjelovic et al., 2022; Chen et al., 2023). Catalase is activated to protect cells from reactive oxygen species. Previous studies have also reported catalase overexpression in the proteome analysis of microorganisms that degrade polyethylene (Zadjelovic et al., 2022). In contrast, the oxidation process, which is the first step in the biological degradation of plastics, relies on oxygenase enzymes that require reactive oxygen species (ROS) for their activation. Highly reactive species, such as superoxide radicals and hydrogen peroxide, can either directly attack and oxidize plastics or catalyze the reactions of oxygenases. *P. aeruginosa*, used in this study, contains several oxygenases, including phenol hydroxylase, alkane oxygenase, and cytochrome P450 monooxygenase, all of which can oxidize structures like the CH-CH groups present in polystyrene (Cafaro et al., 2004; Liu et al., 2014; Price et al., 2022). Therefore, catalase may play a competitive role with these oxygenases in the initial oxidation stage of plastic degradation. Future research could focus on suppressing catalase expression to enhance oxygenase-mediated oxidation.

**Table 1:** The protein identified through LC–MS sequencing and profiling from the distinctive band observed in *P. aeruginosa* that degraded polystyrene.

Location	Protein	Accession number
Cytoplasm	Catalase KatA	tr|V6AKY4|V6AKY4_PSEAI
Cytoplasm	Chaperonin GroEL	tr|A0A9Q9JWE5|A0A9Q9JWE5_PSEAI
Cytoplasm	Glutamine synthetase	tr|V6AND2|V6AND2_PSEAI
Cytoplasm	Isocitrate lyase	tr|V6ADR4|V6ADR4_PSEAI
Extracellular	Neutral metalloproteinase	tr|I6R951|I6R951_PSEAI

Chaperonin GroEL may be expressed to maintain protein folding under stress conditions (Kumar et al., 2015). Using plastic as a carbon source for growth can be considered a stress condition due to the limited availability of nutrients, which likely induces the expression of the associated genes. Isocitrate lyase is a key enzyme in the glyoxylate cycle and is essential for energy metabolism (Prasad Maharjan et al., 2005). Given its activation in processes that metabolize molecules such as lipids and the known involvement of plastic intermediates in lipid metabolism, its expression suggests a pivotal role in cellular metabolism induced by plastics (Zadjelovic et al., 2022). Proteins identified in the extracellular space include neutral metalloproteinase, which is a protein-degrading enzyme involving metal in its reaction (Supplementary Table S4; Wu and Chen, 2011). Metalloproteinase is an enzyme whose role in plastic degradation has not yet been established. However, as a type of hydrolase, neutral metalloproteinase may be involved in the depolymerization of polystyrene (Zhang et al., 2022). Therefore, metalloproteinase is a promising candidate for direct involvement in polystyrene degradation. Future studies should focus on cloning, expressing, and purifying this gene to evaluate its degradation activity not only on polystyrene but also on other polyolefin plastics, such as polyethylene and polypropylene.

## 4 Conclusion

In this study, the biological degradation of PS was analyzed following transformation from the solid to liquid state. The results revealed a significantly higher degradation rate for PS in the liquid state compared to the results from previous studies and solid-state reactions. XPS and FT-IR analyses of the degraded PS confirmed that more pronounced oxidation occurred in the liquid-state, leading to an increase in oxygen species and the formation of C-O, C=O, and –OH groups. Additionally, GPC and TGA confirmed that changes in the molecular weight and thermal stability of the plastics occurred when they reacted in the liquid phase. In summary, the characteristics of plastics, which are insoluble in water owing to their hydrophobic nature, appear to be a major limiting factor for their degradation rate.

To overcome this limitation, for effective degradation, we propose the transformation of plastics into a liquid state. The solvent chosen for this approach should exhibit the following characteristics: (1) ability to dissolve the plastic, (2) high solubility to dissolve a substantial amount of plastic with minimal solvent usage, (3) no interference with biological degradation capabilities (no toxicity to microorganisms or enzyme modification), and (4) nonvolatility (to prevent the plastic from solidifying again during the cultivation process). This study explored the potential to accelerate biological degradation reactions by modifying the characteristics of plastics. However, an assessment of its commercialization potential was not performed. While the study focused on analyzing the degraded state of the plastic, the final products, including degradation byproducts, were not investigated. Therefore, future research should focus on identifying suitable solvents and optimizing cultivation conditions to further enhance the biological degradation of plastics. By selecting effective solvents, economic feasibility can be evaluated. Additionally, qualitative and quantitative analysis of the degradation products will provide deeper insights into how plastic characteristics affect biological degradation. This approach could ultimately contribute to the development of more sustainable waste management technologies.

## Data Availability

The original contributions presented in the study are included in the article/Supplementary material, further inquiries can be directed to the corresponding author.
