# Supplementary material for: Enhancing the oxidation of polystyrene through a homogeneous liquid degradation system for effective microbial degradation
Source: Front Microbiol. 2024 Nov 28;15:1509603. doi: 10.3389/fmicb.2024.1509603 (PMC11636969; doi:10.3389/fmicb.2024.1509603)
Supplement: Supplementary file 1 [file Supplementary_file_1.docx]

**Supplementary Information**

**Enhancing the Oxidation of Polystyrene Through a Homogeneous Liquid Degradation System for Effective Microbial Degradation**

**Hong Rae Kim^a,1^, Hye Yeon Koh^a,1^, Hyeyoung Shin^a^, Dong-Eun Suh^a^, Sukkyoo Lee^b^, Donggeon Choi^a,^ ***

^a^*Department of Research and Development, Repla Inc., Suwon 16679, Republic of Korea*

^b^*Department of Brain Sciences, Daegu Gyeonbuk Institute of Science and Technology, Daegu 42988, Republic of Korea*

^1^ H.R. Kim and H.Y. Koh equally contributed to the work

***Corresponding author**

Mailing address: Department of Research and Development, Repla Inc., 237, Yeongtong-ro, Yeongtong-gu, Suwon-si, Gyeonggi-do, Republic of Korea (16679), Phone: (+82) 31-895-4310. Fax: (+82) 70-4667-0230. E-mail: [dgchoi@repla.co.kr](mailto:dgchoi@repla.co.kr)

**Materials and Methods**

**Text S1. In-gel trypsin digestion and Mass Spectrometry**

Band was collected from 1D gel. The gel plugs were destained in 50% acetonitrile (ACN), washed three times with distilled water, and incubated with 50 mM ammonium bicarbonate (ABC). Reduction and alkylation were performed using 1 M dithiothreitol (GE Healthcare, Chicago, IL, USA) and 1 M iodoacetamide (Sigma-Aldrich, St. Louis, MO, USA) at room temperature for 1 h. In-gel digestion was performed using 20 ng/μL sequencing grade trypsin (Promega Corporation, Madison, WI, USA) in 50 mM ammonium bicarbonate, pH 7.8, and incubated at 37°C for 16 h. By adding 200 μL of 50% ACN solvent, the hydrolyzed peptide is recovered through dehydration reaction of gel. Sample clean-up was carried out using C18 spin tips (Thermo Fisher Scientific, Waltham, MA, USA). The column was activated using 100% ACN and samples were loaded on to the column after washing thoroughly to remove traces of ACN. The peptides on the column eluted with an organic solution containing at 80% ACN. The samples were completely dried in a SpeedVac (Thermo Fisher Scientific, Waltham, MA, USA) and stored at -80°C till injection into the mass spectrometer.

The samples were analyzed on a Triple TOF^®^ 5600+ (AB SCIEX, Seoul, Republic of Korea) with Protein pilot and peak view software. Data were acquired in positive reflector mode over a mass range of 300 to 2,500 m/z using external calibration spots with TOF/TOF calibration mixture. Protein pilot 4.0 was used to process MS and MS/MS spectra to submit the peak list to the MASCOT v1.9 search engine (<http://www.matrixscience.com>: Matrix Science) for peptide identification against the Universal Protein Resource (UniProt) databases. The unused protein score is the total amount of unique peptide evidence associated with a given protein, with a confidence score of > 2.0 used as the qualifying criteria. This corresponds to a peptide confidence level of 95%.

**Table S1.** *P. aeruginosa* proliferating using polystyrene foam as a carbon source.

|  | **D0** | **D7** | **D14** | **D21** | **D28** |
| --- | --- | --- | --- | --- | --- |
| Colony-forming unit | 9.85E+05 | 1.67E+07 | 4.40E+07 | 5.97E+07 | 9.43E+07 |

**Table S2.** Polystyrene surface elemental analysis using XPS.

| **Polystyrene** | ***P. aeruginosa*** | **C** | **O** | **N** | **P** | **S** |
| --- | --- | --- | --- | --- | --- | --- |
| Solid | X | 94.84 | 4.68 | 0.34 | 0.08 | 0.06 |
| Solid | O | 89.21 | 8.00 | 2.45 | 0.15 | 0.19 |
| Liquid | X | 94.92 | 4.17 | 0.59 | 0.20 | 0.13 |
| Liquid | O | 82.95 | 15.51 | 0.68 | 0.61 | 0.26 |

**Table S3.** The cytoplasmic protein identified through LC-MS sequencing and profiling from the distinctive band observed in *P. aeruginosa* that degraded polystyrene.

| **NO** | **Unused protein Score** | **Sequence coverage (%)** | **Name** | **Remarks** |
| --- | --- | --- | --- | --- |
| 1 | 115.0 | 77.5 | Chaperonin GroEL | Major |
| 2 | 110.0 | 81.1 | Catalase KatA | Major |
| 3 | 100.7 | 74.8 | Glutamine synthetase | Major |
| 4 | 96.5 | 67.6 | Isocitrate lyase | Major |
| 5 | 59.3 | 56.7 | Small ribosomal subunit protein bS1 | Minor |
| 6 | 59.2 | 57.5 | Electron transfer flavoprotein-ubiquinone oxidoreductase | Minor |
| 7 | 45.7 | 49.7 | Acyl-CoA dehydrogenase | Minor |
| 8 | 43.3 | 40.3 | Quinoprotein ethanol dehydrogenase | Minor |
| 9 | 35.1 | 42.9 | Glucans biosynthesis protein G | Minor |
| 10 | 30.1 | 35.7 | Phosphoenolpyruvate carboxykinase (ATP) | Minor |
| 11 | 27.6 | 15.9 | DNA-directed RNA polymerase subunit beta' | Minor |
| 12 | 24.0 | 27.1 | Polyribonucleotide nucleotidyltransferase | Minor |
| 13 | 23.8 | 33.1 | Dihydrolipoyl dehydrogenase | Minor |
| 14 | 22.8 | 28.4 | Amidohydrolase | Minor |
| 15 | 21.6 | 32.8 | Chaperone protein DnaK | Minor |
| 16 | 20.5 | 20.8 | Acyl-CoA dehydrogenase | Minor |
| 17 | 20.1 | 29.6 | NAD+ dependent aldehyde dehydrogenase ExaC | Minor |
| 18 | 19.9 | 17.4 | DNA-directed RNA polymerase subunit beta | Minor |
| 19 | 19.1 | 31.8 | 2,3-bisphosphoglycerate-independent phosphoglycerate mutase | Minor |
| 20 | 17.6 | 28.4 | 2-isopropylmalate synthase | Minor |
| 21 | 17.3 | 20.1 | Aconitate hydratase B | Minor |
| 22 | 17.0 | 23.9 | Urease subunit alpha | Minor |
| 23 | 16.0 | 21.0 | Probable acyl-CoA dehydrogenase | Minor |
| 24 | 15.7 | 18.6 | Elongation factor G | Minor |
| 25 | 14.0 | 23.7 | Glutamine--tRNA ligase/YqeY domain fusion protein | Minor |
| 26 | 13.6 | 28.3 | Long-chain-fatty-acid-CoA ligase | Minor |
| 27 | 12.8 | 25.7 | Arginine--tRNA ligase | Minor |
| 28 | 12.2 | 18.3 | Flp pilus assembly protein TadD2 | Minor |
| 29 | 10.3 | 17.9 | Periplasmic dipeptide transport protein | Minor |
| 30 | 10.0 | 10.1 | Penicillin acylase family protein | Minor |
| 31 | 10.0 | 17.7 | Aminopeptidase | Minor |
| 32 | 10.0 | 15.9 | Pyruvate kinase | Minor |
| 33 | 8.3 | 11.6 | Sulfite reductase | Minor |
| 34 | 8.0 | 19.7 | Putative acyl-CoA dehydrogenase | Minor |
| 35 | 8.0 | 9.5 | Glutamate--cysteine ligase | Minor |
| 36 | 7.2 | 13.4 | Dihydroxy-acid dehydratase | Minor |
| 37 | 7.1 | 17.4 | Cytochrome c domain-containing protein | Minor |
| 38 | 6.7 | 13.0 | PHA-synthase2 | Minor |
| 39 | 6.0 | 10.3 | Elongation factor Tu | Minor |
| 40 | 6.0 | 10.0 | Putative hemolysin activation/secretion protein | Minor |
| 41 | 5.4 | 10.5 | Bifunctional purine biosynthesis protein PurH | Minor |
| 42 | 5.2 | 13.4 | Succinate--CoA ligase [ADP-forming] subunit beta | Minor |
| 43 | 4.7 | 13.4 | Periplasmic dipeptide transport protein | Minor |
| 44 | 4.6 | 8.2 | Probable carbamoyl transferase | Minor |
| 45 | 4.1 | 11.4 | Probable acyl-CoA dehydrogenase | Minor |
| 46 | 4.0 | 9.1 | Outer membrane porin F | Minor |
| 47 | 4.0 | 6.8 | Biosynthetic arginine decarboxylase | Minor |
| 48 | 4.0 | 4.6 | Urocanate hydratase | Minor |
| 49 | 4.0 | 6.9 | Dihydrolipoyllysine-residue acetyltransferase component of pyruvate dehydrogenase complex | Minor |
| 50 | 4.0 | 11.4 | Amidase family protein | Minor |
| 51 | 3.6 | 4.6 | Phosphogluconate dehydratase | Minor |
| 52 | 2.1 | 7.8 | Methyl-accepting chemotaxis protein | Minor |
| 53 | 2.1 | 19.8 | Alkyl hydroperoxide reductase C | Minor |
| 54 | 2.0 | 5.1 | Phosphoenolpyruvate synthase | Minor |
| 55 | 2.0 | 45.1 | 30S ribosomal protein | Minor |
| 56 | 2.0 | 20.0 | Aldehyde dehydrogenase | Minor |

**Table S4.** The extracellular protein identified through LC-MS sequencing and profiling from the distinctive band observed in *P. aeruginosa* that degraded polystyrene.

| **NO** | **Unused protein Score** | **Sequence coverage (%)** | **Name** | **Remarks** |
| --- | --- | --- | --- | --- |
| 1 | 64.6 | 46.2 | Neutral metalloproteinase | Major |
| 2 | 13.1 | 29.5 | Flagellin | Minor |
| 3 | 10.4 | 13.3 | Putative aminopeptidase | Minor |
| 4 | 9.7 | 9.6 | Quinoprotein ethanol dehydrogenase | Minor |
| 5 | 9.1 | 15.1 | Outer membrane porin F | Minor |
| 6 | 8.3 | 20.5 | Isocitrate lyase | Minor |
| 7 | 7.2 | 11.9 | Porin | Minor |
| 8 | 7.0 | 10.6 | Probable outer membrane protein | Minor |
| 9 | 5.8 | 7.9 | Elongation factor G | Minor |
| 10 | 4.3 | 20.2 | Glutamate/aspartate periplasmic-binding protein | Minor |
| 11 | 4.0 | 9.5 | Poly(ethylene terephthalate) hydrolase | Minor |
| 12 | 4.0 | 13.7 | N-acetylmuramoyl-L-alanine amidase domain-containing protein | Minor |
| 13 | 4.0 | 5.1 | Acyl-CoA dehydrogenase family member 10 | Minor |
| 14 | 4.0 | 9.3 | Putative isomerase | Minor |
| 15 | 2.4 | 4.3 | Type VI secretion protein | Minor |
| 16 | 2.0 | 45.0 | Neutral metalloproteinase (Fragment) | Minor |
| 17 | 2.0 | 3.8 | Dihydrolipoyl dehydrogenase | Minor |


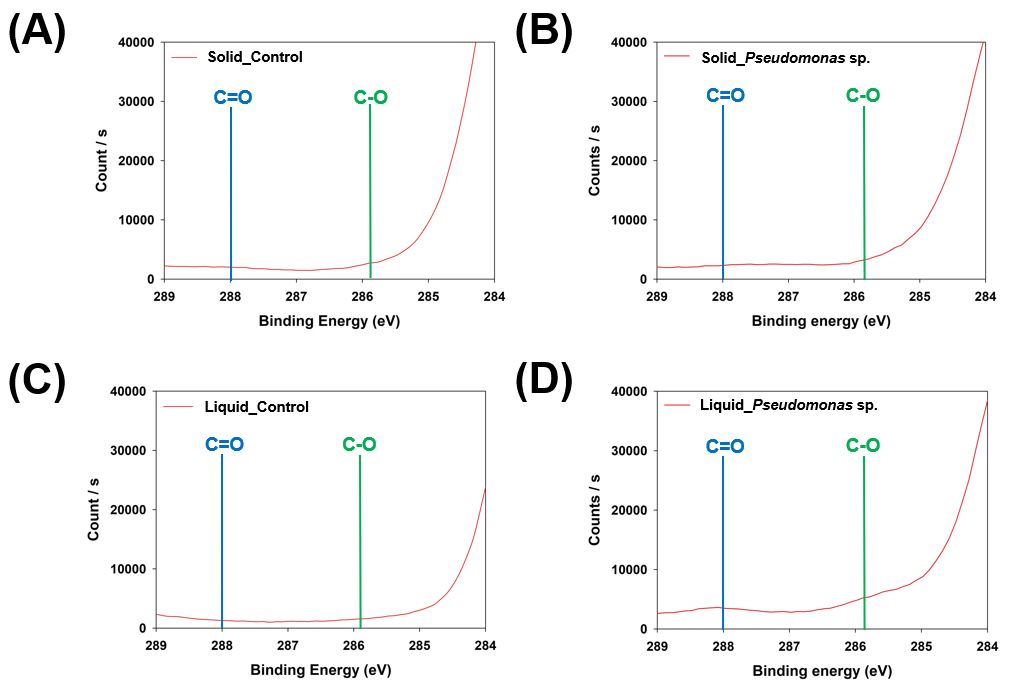


**Figure S1. Analysis of Carbon Binding Energy in Polystyrene using XPS.** (A) To clearly identify the binding energies of C-O and C=O, 284eV – 289eV region was magnified. Control group cultured with *P. aeruginosa* in the solid state. (B) Polystyrene cultured with *P. aeruginosa* in the solid state. (C) Control group cultured with *P. aeruginosa* in the liquid state. (D) Polystyrene cultured with *P. aeruginosa* in the liquid state.


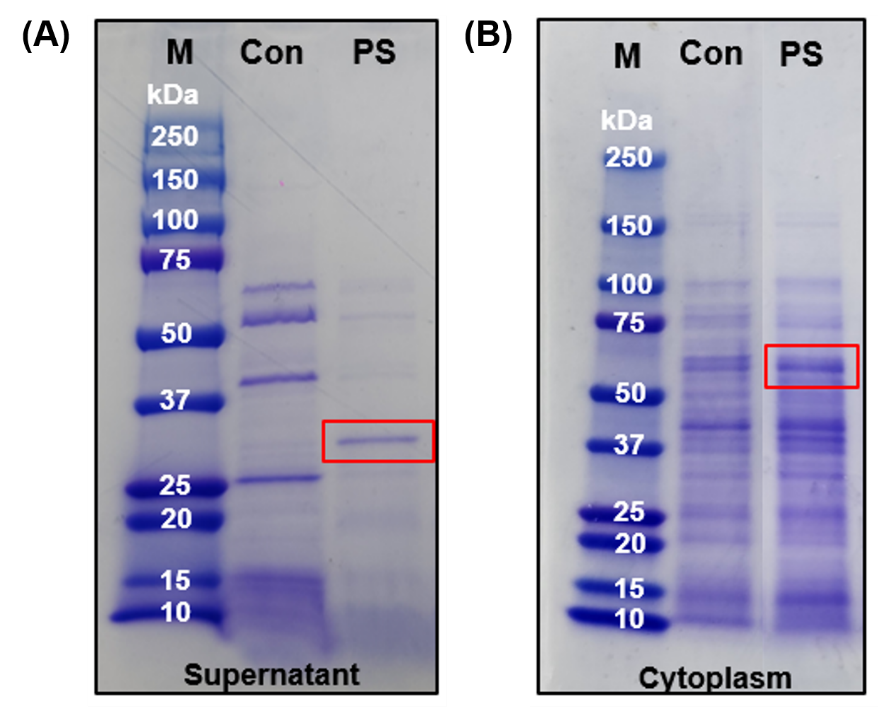


**Figure S2. Changes in Protein Expression Patterns During Polystyrene Degradation.** (A) Extracellular proteins. (B) Cytoplasmic proteins. M: Marker, Con: Proteins of *P. aeruginosa* cultured with ethyl ester oil., PS: Proteins of *P. aeruginosa* cultured with polystyrene-dissolved ethyl ester oil.
